# Predicting EQ-5D-5L crosswalk from the PROMIS-29 profile for the United Kingdom, France, and Germany

**DOI:** 10.1186/s12955-020-01629-0

**Published:** 2020-12-17

**Authors:** Christoph Paul Klapproth, J. van Bebber, C. J. Sidey-Gibbons, J. M. Valderas, A. Leplege, M. Rose, F. Fischer

**Affiliations:** 1 Department of Psychosomatic Medicine, Center for Internal Medicine and Dermatology, Charité - Universitätsmedizin Berlin, Charitéplatz 1, 10117 Berlin, Germany; 2 Department of Symptom Research, MD Anderson Cancer Center, University of Houston, Houston, TX USA; 3 Health Services and Policy Research Group, University of Exeter, Exeter, UK; 4 NIHR Peninsula Collaboration for Leadership in Applied Health Research and Care, Exeter, UK; 5 APEMAC, EA 4360, Paris Descartes University, Paris, France; 6 Département d’Histoire et de Philosophie des Sciences, Laboratoire SPHERE, UMR 7219, CNRS-Université Paris Diderot - Sorbonne Paris Cité, Paris, France; 7 Department of Quantitative Health Sciences, University of Massachusetts Medical School, Worcester, USA

## Abstract

**Background:**

EQ-5D health state utilities (HSU) are commonly used in health economics to compute quality-adjusted life years (QALYs). The EQ-5D, which is country-specific, can be derived directly or by mapping from self-reported health-related quality of life (HRQoL) scales such as the PROMIS-29 profile. The PROMIS-29 from the Patient Reported Outcome Measures Information System is a comprehensive assessment of self-reported health with excellent psychometric properties. We sought to find optimal models predicting the EQ-5D-5L crosswalk from the PROMIS-29 in the United Kingdom, France, and Germany and compared the prediction performances with that of a US model.

**Methods:**

We collected EQ-5D-5L and PROMIS-29 profiles and three samples representative of the general populations in the UK (n = 1509), France (n = 1501), and Germany (n = 1502). We used stepwise regression with backward selection to find the best models to predict the EQ-5D-5L crosswalk from all seven PROMIS-29 domains. We investigated the agreement between the observed and predicted EQ-5D-5L crosswalk in all three countries using various indices for the prediction performance, including Bland–Altman plots to examine the performance along the HSU continuum.

**Results:**

The EQ-5D-5L crosswalk was best predicted in France (nRMSE_FRA_ = 0.075, nMAE_FRA_ = 0.052), followed by the UK (nRMSE_UK_ = 0.076, nMAE_UK_ = 0.053) and Germany (nRMSE_GER_ = 0.079, nMAE_GER_ = 0.051). The Bland–Altman plots show that the inclusion of higher-order effects reduced the overprediction of low HSU scores.

**Conclusions:**

Our models provide a valid method to predict the EQ-5D-5L crosswalk from the PROMIS-29 for the UK, France, and Germany.

## Key points

- We provide mapping from PROMIS-29 profile to EQ-5D-5L crosswalk in the United Kingdom, France, and Germany.
- Due to the country specificity of health state utility, mapping algorithms for health state utility should not be generalized across countries.
- The application of polynomial regression models that account for non-linearity improves the prediction performance, in particular for poorer health states.
- The application of foreign models should be avoided.

## Background

Quality-adjusted life years (QALYs) are routinely used in cost-utility analyses (CUA) to evaluate the economic effectiveness of health care innovations or interventions [1]. QALYs are of particular importance in health technology assessments (HTAs) [2]. For example, the National Institute of Health and Clinical Excellence (NICE) in England and Wales has endorsed QALYs to compare health care interventions from an economic perspective [1]. In light of budget constraints in publicly funded health care systems, QALYs serve as a benchmark for the allocation of scarce resources in a way that maximizes utility to individuals and to society [2].

A QALY is defined as the product of the number of life years and a health state utility (HSU) score that represents the value of a particular health state. HSU values can at best achieve a value of 1 (full health). A value of 0 is considered dead and health states with a negative value are considered worse than dead. Individual HSU scores are patient-reported, generic, preference-based measures of health-related quality of life (HRQoL) [3]. The most frequently used generic HRQoL measure is the EuroQoL EQ-5D-5L crosswalk differentiating 3125 (i.e., 5^5^) health states. The EQ-5D-5L crosswalk is the default HSU score for economic evaluations demanded by HTA agencies such as NICE [4–7].

The Patient Reported Outcome Measurement Information System (PROMIS), on the other hand, is increasingly used internationally to measure clinical and condition-specific, non-preference HRQoL for its favourable psychometric properties: high validity, high reliability, high precision, and flexible administration [8, 9]. PROMIS is a common metric for a large variety of different health domains, aiming at comprehensive assessment, standardization and integration of different measures and items. It constitutes a collection of generic and condition-specific, non-preference-based patient reported outcome measures (PROMs) that have been developed using item response theory (IRT) [10]. For each PROM, so-called item banks have been developed comprising items that are highly informative regarding the PROM to be measured and that do not function substantially different across the most prominent demographic groups (e.g., women and men) [11, 12]. These item banks can be used to develop tailored short forms or for computerized adaptive testing (CAT) [13]. PROMIS overcomes significant limitations of legacy instruments such as ceiling effects and is, being translated to many languages and showing invariance to nationality, becoming the international reference measurement approach to PROMs [9, 14–16].

For economic evaluations, the preference-based EQ-5D-5L crosswalk is best obtained directly using the EQ-5D-5L questionnaire. If direct assessment is not available, a common strategy is to estimate HSU scores by using a mapping algorithm from a non-preference-based PROM such as PROMIS [14, 17–20]. Little consensus exists on which mapping method is the most appropriate. In a recent systematic review, 147 studies mapping the EQ-5D were identified [17]. In more than 75% ordinary least squares (OLS) linear regression was used. Although OLS linear regression showed robust results compared to alternative methods, it has several drawbacks [21, 22]: First, predicted HSU scores may fall outside the possible range of the metric (i.e., values greater than one). Second, the relationship between non-preference-based PROM and HSU might be non-linear, meaning that the impact of health domains differs across the HSU continuum [22].

As PROMIS is increasingly used in clinical, non-preference HRQoL measurement and the EQ-5D-5L crosswalk is the required HSU for economic evaluations, developing a mapping between these two would open the perspective to use PROMIS for economic evaluations. As both are multidimensional generic HRQoL measures covering similar dimensions or domains (EQ-5D mobility and EQ-5D self-care versus PROMIS physical function, EQ-5D pain/discomfort versus PROMIS pain interference, EQ-5D anxiety/depression versus PROMIS anxiety or PROMIS depression, EQ-5D usual activities versus PROMIS ability to participate in social roles and activities), we can reasonably assume conceptual overlap, as previous mappings have as well [19, 20].

Mapping PROMIS to EQ-5D-5L crosswalk also opens a perspective for the use of other PROMs in economic evaluations: Because of its invariance property, PROMIS domains can also be measured using items from a different condition-specific measure that is anchored to the PROMIS metrics. For example, items from self-reported anxiety measured by MASQ, PANAS and GAD-7 are anchored on the PROMIS Anxiety metric [23]. Items from the BDI-2, CES-D, and PHQ-9, measuring depression, are anchored on the PROMIS Depression metric [24]. Therefore, mapping from PROMIS T-scores to EQ-5D-5L crosswalk enables the mapping of a broad range of PROMs to the EQ-5D-5L crosswalk via PROMIS.

Using OLS linear regression on US data, Revicki (2009) estimated a model to predict the former EQ-5D version, the EQ-5D-3L index value, from five PROMIS T-scores [19]: physical function, fatigue, pain interference, anxiety, and depression. For this PROMIS domain model, Revicki reports that approximately 57% (adjusted R^2^) of the variance in EQ-5D-3L index value can be explained by the variables in the model, and the intraclass correlation coefficient (ICC) is 0.73. Furthermore, 95% of all the residuals are between − 0.20 (2.5%) and 0.15 (97.5%). The relatively small width of these so-called empirical limits of agreement (LoA) is indicative of an appropriate fitted model. However, Revicki also reported that the model does not work very well for low levels of health (EQ-5D-3L index value < 0.40). Revicki used the EQ-5D-3L questionnaire and applied the US EQ-5D-3L value set by Shaw (2005) [25]. As health preferences differ between countries, the EQ-5D-3L index value is country-specific [26, 27]. Revicki’s model can therefore only be used to predict the EQ-5D-3L index value from PROMIS in the US.

Therefore, the primary aim of this study is to develop mapping functions from PROMIS-29 to the EQ-5D-5L crosswalk for the UK, France, and Germany so that PROMIS can be used for economic valuations in these countries. For each health domain, we explored the form of its relationship with the EQ-5D-5L crosswalk and examined whether these relationships would be the same across the three countries under investigation. Also, we aimed at improving prediction performance by including higher order coefficients. Furthermore, we investigated whether the optimal models would be structurally equivalent across countries and compared prediction performance of our models to Revicki’s model.

## Methods

### Samples

Data were collected online by an independent polling company (Ipsos) in April and May 2015. Quota sampling was employed to obtain samples representative of the general population with respect to sex, age, occupation, region, and population density of the UK (n = 1509), France (n = 1501), and Germany (n = 1502). Sample weights were calculated using the random iterative method (RIM) to match the latest data available in each country (census 2011 for the UK and Germany, census 2012 for France).

Participation in our general population samples was voluntary and data protection laws obeyed by Ipsos. If a respondent chose to drop out at some point, the data given until that point was not included. As skipping items was not possible, there were no missing data.

### Measures

#### PROMIS domains and item banks

We used the PROMIS-29 v2.0 Profile to assess seven core domains of health, each assessed with four items: physical function, fatigue, pain interference, anxiety, depression, sleep disturbance, and the ability to participate in social roles and activities (referred to as participation in the remainder of this article) plus the visual analogue scale (VAS) expressing pain intensity on a scale ranging from 0 to 10 [28]. PROMIS-29 has, compared to other short forms, enough items to achieve a sufficient degree of precision while maintaining a reasonable response burden. Items are measured on five levels (e.g. “never”, “rarely”, “sometimes”, “often”, “always” or “not at all”, “a little bit”, “somewhat”, “quite a bit”, “very much”) and refer to the past 7 days (except physical function). Answers yield a number from one to five, which, once fed into the online PROMIS converter (http://www.healthmeasures.net/score-and-interpret/calculate-scores), give one correspondent PROMIS T-Score (M = 50 ± SD = 10) per domain with the US general population as a reference. Note that due to the invariance property of IRT, T-Scores obtained from the PROMIS-29 are on the same metric as the scores Revicki used in his analysis, though these scores were generated using different items. For desirable constructs (e.g., physical function), higher T-scores indicate better health, whereas for undesirable domains (e.g., depression), higher T-scores indicate poorer health states.

The psychometric properties of the PROMIS-29 profile, including evidence of construct and criterion validity, have been reported elsewhere [29–32]. An earlier analysis of the data used in this study revealed that scores on the seven health domains of the PROMIS-29 are measurement invariant across the UK, France, and Germany except for one item [33].

#### EQ-5D-5L crosswalk value set

The EuroQoL EQ-5D is a standardized patient-reported HRQoL questionnaire, measuring five health dimensions of health: mobility, self-care, usual activities, pain/discomfort, and anxiety/depression. Its original version, the EQ-5D-3L, differentiates 3 levels per domain, defining 3^5^ or 243 health states. Its revised version, the EQ-5D-5L, has five levels: “No problems” (or 1), “Slight problems” (2), “Moderate problems” (3), “Severe problems” (4), and “Extreme problems” (5), defining 5^5^ or 3125 different health states. We chose the EQ-5D-5L questionnaire because it can differentiate more health states and is more sensitive. Each health state is assigned a HSU by different value sets, reflecting the preferences of the general population in the respective countries. For many countries, there is not yet a value set for the 5L version. An EQ-5D-5L crosswalk value set was developed for the purpose of using 3L value sets for health states described by the 5L version. We used these EQ-5D-5L crosswalk value sets as they are available for all three countries of our samples [4, 26, 34, 35].

The maximum HSU for the best health state of 11111 is 1.00 or “full health” while 0.00 is considered “dead”. The minimum HSU of the worst health state of 55555 is negative, considered “worse than dead”: − 0.594 in the UK, − 0.530 in France, and − 0.205 in Germany [26].

### Statistical analysis

#### Relationships among individual health domains and health state utility across the UK, France, and Germany

To obtain a first impression of the form of the relationships among individual health domains and HSU and to judge whether the relationships are stable across the three countries under investigation, we plotted the seven domain scores against HSU in the UK, France, and Germany.

#### Optimal models for predicting health state utility in the three countries

We applied stepwise regression with backward selection to find the best models to predict the EQ-5D-5L crosswalk for the UK, France, and Germany, starting with full models that incorporated linear, quadratic, and cubic effects for all seven PROMIS-29 domains. We included polynomials up to the third degree as we expected that such polynomials can more flexibly fit the observed data, e.g. in case of nonlinear relationships between predictors and outcome. We used raw polynomials for linear, quadratic and cubic effects in order to obtain coefficients which can be used for prediction independently.

Because sociodemographic factors such as age and sex are known to be useful in predicting HSU, they were also entered as possible predictors [17]. The PROMIS pain intensity VAS was not included as pain is already covered by the pain interference domain, which proved to be superior than the VAS [36]. Also, while all other domains comprise of 4 items, the pain intensity domain within PROMIS-29 has only this single item, not measured on a T-Score metric.

The Bayesian information criterion (BIC) was used to steer the inclusion and exclusion of predictors in the stepwise regression analyses [37]. We chose nRMSE and nMAE as measures of the prediction precision and bias as they are preferred over either R^2^ or BIC used by Revicki [19, 38]. The nRMSE is the normalized root of the sum of the squared residuals between observed and predicted scores and the nMAE is the normalized mean absolute error of the absolute residuals. Both are normalized with respect to the different scale ranges of the EQ-5D-5L crosswalk in the UK, France, and Germany [39–41]. We also determined the width between the 95% empirical limits of agreement and compared them to the 95% theoretical limits of agreement (i.e., ± 1.96 × SD(residuals)). To check the prediction performance along the HSU continuum, Bland–Altman plots were used.

We use cross-validation to check for overfitting [42]. With this in-sample cross-validation technique, the initial dataset is randomly split into 10 subsamples of approximately equal size. One of these subsamples is kept for validation, while the other nine subsamples are used for parameter estimation. This process is repeated ten times, and the results are averaged across repetitions. Overfitting would show when a model’s nRMSE is substantially smaller than the average nRMSE of the models of the 10 subsamples.

We used *R* version 3.4.1, IBM SPSS Statistics version 23, and Microsoft Excel version 15 to run the analyses.

#### Impact of misspecified mapping functions on the prediction performance

To the best of our knowledge, as of December 2020, the mapping function by Revicki was the only one available for predicting the EQ-5D-3L index value from the PROMIS-29 T-scores [19]:

$$\begin{aligned} EQ - 5D & = 1.0266 + 0.0077 \times Physical\,functioning - 0.0021 \times fatigue \\ & \quad - \,0.0040 \times Pain\,interference - 0.0023 \times anxiety - 0.0022 \times depression \\ \end{aligned}$$

We were interested in quantifying the detrimental effect of applying this foreign mapping function to the data collected in Europe. Note that application of Revicki’s model to the data collected in the UK, France and Germany (1) disregards the country specificity of any version of the EQ-5D, (2) does not utilize the potential predictive value of the two PROMIS-29 health domains not used by Revicki, (3) does not take higher-order effects into account, and in combination with the foregoing, (4) disregards country dependency of the form of relationships (i.e., the specific values of the regression coefficients used).

Because we were also interested in which factor is mainly responsible for the differences in prediction performance, we moved stepwise from Revicki’s model to our models as follows: First, we used the five health domains of Revicki’s model, but with regression coefficients optimized towards the data collected in each country separately. Second, we investigated the incremental value of adding either sleep disturbance, participation, or both to the prediction equation. Third, we allowed for incorporation of quadratic and/or cubic effects.

## Results

### Sample characteristics

We only briefly summarize the most important differences between the three samples here. The interested reader is referred to Table 4 (See “Appendix” section) for a comprehensive overview of the marginal distributions of sex, age, educational level, occupational status, and income in the three samples. Participants in the German sample (mean age = 50.0 years old) were slightly older than participants in the French (48.4 years old) and UK samples (47.8 years old). Participants in the German sample were more likely to have a low educational background (23.4%) than participants in the French (7.6%) and UK samples (8.1%). Participants in the French sample were more likely to be unemployed/inactive (48.4%) than participants in the German (41.5%) and UK samples (39.4%).

### Relationships among individual health domains and health state utility across the UK, France, and Germany

**Fig. 1:**
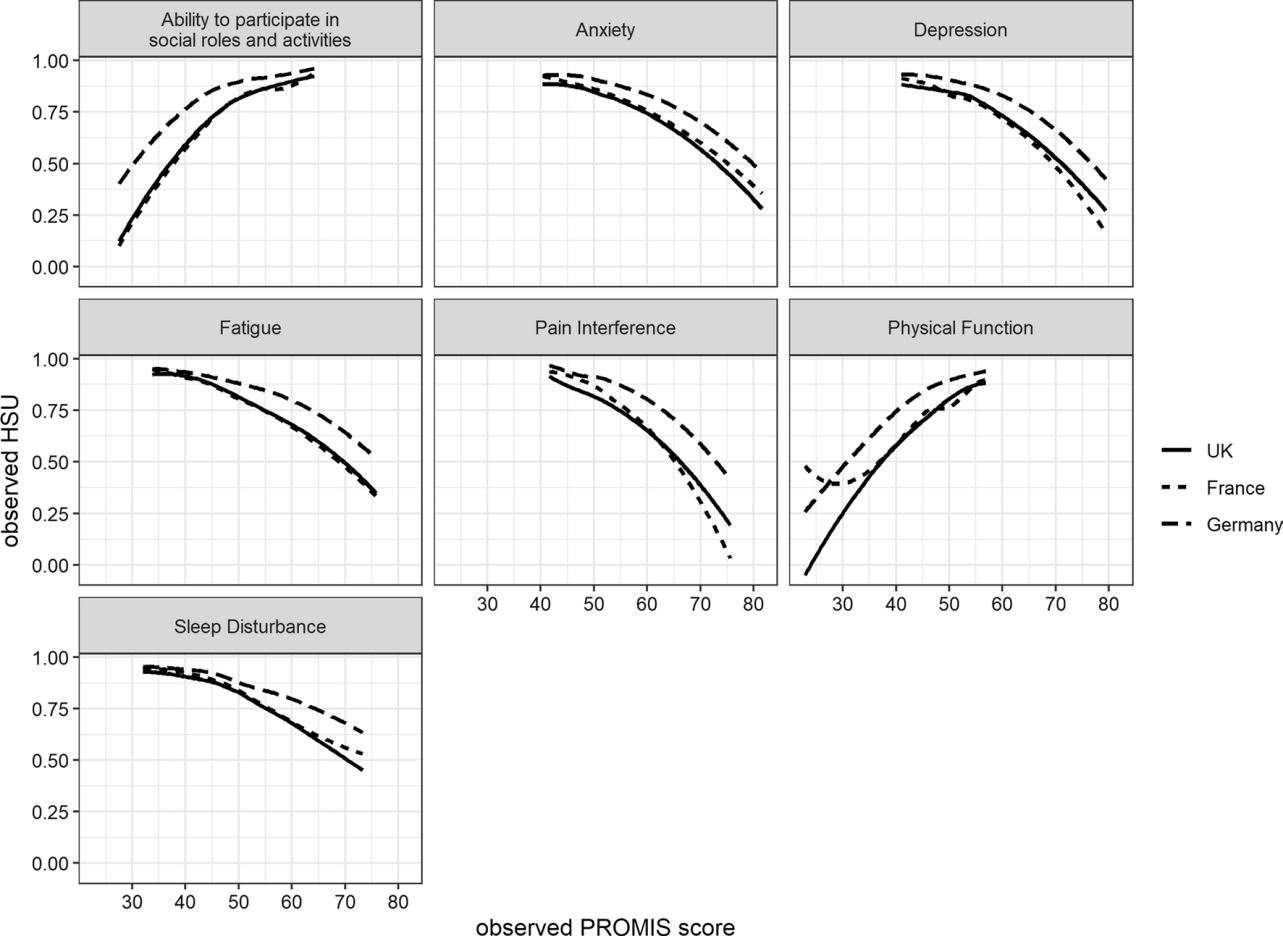
Relationships among the PROMIS domains and health state utility expressed by the EQ-5D-5L crosswalk

The relationships among the seven PROMIS domains and HSU expressed by the EQ-5D score in the three European countries are displayed in Fig. 1.

A number of conclusions can be drawn from Fig. 1. First, with the exception of low levels of physical functioning in France, the relationships among the seven PROMIS domains and HSU are comparable across the three European countries. Second, most of the curves are not simple straight lines and are slightly curvilinear, indicating that changes at severer levels have a greater impact on HSU. Third, all the relationships are in accordance with theoretical expectations. Higher values on the positive PROMIS domains (participation and physical function) correspond to higher HSU values, and higher values on the five negative PROMIS domains correspond with lower HSU values. Fourth, participation and physical function seem to have the strongest relationship with HSU because these curves are the steepest.

### Optimal models for predicting health state utility in the three countries

Recall that we used stepwise regression with backward selection to find optimal models for predicting HSU for the UK, France, and Germany. The primary models thus comprised linear, quadratic, and cubic effects for each PROMIS domain plus effects for age and sex. Effects that did not significantly improve the prediction performance were sequentially removed from these models. The coefficients of the final models to optimally estimate the EQ-5D-5L crosswalk from PROMIS-29 for the UK, France, and Germany can be found in Table 1.

**Table 1:** Coefficients of the optimal models for the United Kingdom, France, and Germany

	UK			France			Germany		
	Regression coefficient	Standardized regression coefficient	SE	Regression coefficient	Standardized regression coefficient	SE	Regression coefficient	Standardized regression coefficient	SE
Constant	2.288E−0		7.874E−1	2.910E−0		5.665E−1	− 1.181E−0		3.047E−1
Age	9.590E−4	0.069	2.032E−4	− 1.372E−3	− 0.107	1.903E−4			
Anxiety	1.120E−2	0.499	2.951E−3						
Pain interference	− 1.773E−1	− 7.479	4.27E−2						
Physical function	5.354E−2	1.881	5.24E−3	− 3.027E−1	− 9.807	3.202E−2			
Depression							7.425E−3	0.404	1.664E−3
Participation	1.334E−2	0.573	4.027E−3	9.415E−2	3.719	2.660E−2	8.834E−2	4.915	1.963E−2
Anxiety^a^	− 1.227E−4	− 0.604	2.758E−5						
Pain interference^a^	3.042E−3	13.970	7.651E−4	2.122E−4	0.900	4.059E−5			
Physical function^a^	− 4.853E−4	− 1.566	5.544E−5	7.506E−3	22.864	7.839E−4	5.596E−4	2.581	6.114E−5
Sleep disturbance^a^				− 2.390E−5	− 0.088	4.542E−6	− 1.763E−5	− 0.097	3.415E−6
Participation^a^	− 1.061E−4	− 0.460	3.785E−5	− 1.706E−3	− 7.104	5.465E−4	− 1.733E−3	− 9.850	4.073E−4
Anxiety^b^							− 1.480E−7	− 0.070	5.293E−8
Depression^b^	− 3.453E−7	− 0.145	5.665E−8	− 3.487E−7	− 0.121	5.494E−8	− 8.951E−7	− 0.421	1.991E−7
Fatigue^b^				− 2.456E−7	− 0.088	5.782E−8			
Pain interference^b^	− 1.769E−5	− 6.852	4.460E−6	− 3.697E−6	− 1.270	5.046E−7	− 7.808E−7	− 0.421	4.198E−8
Sleep disturbance^b^	− 1.860E−7	− 0.059	5.000E−8						
Physical function^b^				− 5.805E−5	− 12.841	6.167E−6	− 6.865E−6	− 2.300	8.279E−7
Participation^b^				1.026E−5	3.471	3.670E−6	1.113E−5	4.998	2.763E−6

Coefficients are displayed as negative exponentials with four digits, beginning with the first non-zero digit of the coefficient. HSU is expressed on a scale ranging from − 0.594 (UK), − 0.53 (France), and − 0.205 (Germany) to 1, and the PROMIS domains are expressed as T-scores (M = 50). All the coefficients displayed differ significantly from zero at *p* < 0.01

The (unstandardized) regression coefficients of Table 1 can be used to compute the EQ-5D-5L crosswalk from the PROMIS T-scores: EQ-5D = Constant + Coefficient (Age) × Age + Coefficient (Anxiety) × T-score (Anxiety) + ⋯ + Coefficient (Participation^3^) × (T-score (Participation))^3^. However, interpretation of the regression coefficients needs to take into account two specifics of polynomial regression models.

First, the regression coefficients of the higher-order effects appear to be much smaller than those for the linear effects, as the values of the predictor variables (with M = 50) are taken to the power of two for the quadratic effects (M^2^ = 2,500) and to the power of three for the cubic effects (M^3^ = 125,000). Hence, coefficients have a substantially larger impact on the scale of the criterion.

Second, the single standardized regression coefficients shown in Table 1 should not be used to infer the form of the relationship between the individual health domains and the EQ-5D-5L crosswalk because we have up to three effects (linear, quadratic, and cubic) in each health domain, and the relationship thus must be described by the summed effect of all three effects. Furthermore, not all coefficients are in agreement with Fig. 1 which plotted the relationship of a single health domain to the EQ-5D-5L crosswalk, irrespective of the values in all the other health domains. Instead, the regression coefficients are optimal given the effect of all the other effects already taken into account (stepwise procedure), which also explains why the final models in the three countries are so different. Age, for example, has a positive effect on HSU in the UK, a negative effect on HSU in France, and no effect on HSU in Germany. Although out of the 23 possible predictors twelve (UK and France) and ten (Germany) were kept in the final models, only four effects were consistently chosen across countries: the linear effect of participation, the quadratic effect of physical functioning, and cubic effects of depression and pain interference.

The prediction performance of these models is summarized in Table 2. HSU expressed by the EQ-5D-5L crosswalk can be best mapped from the PROMIS-29 in France (nRMSE_FRA_ = 0.075, nMAE_FRA_ = 0.052), followed by the UK (nRMSE_UK_ = 0.076, nMAE_UK_ = 0.053) and Germany (nRMSE_GER_ = 0.079, nMAE_GER_ = 0.051). Furthermore, for all three countries, the widths of the empirical limits of agreement are always smaller than the widths of the theoretical limits of agreement. All models were confirmed by tenfold cross-validation, having a marginally smaller nRMSE and nMAE compared the mean nRMSE and mean nMAE, respectively, of the 10 models of the cross-validation subsamples.

**Table 2:** Prediction performance of the optimal models for the United Kingdom, France, and Germany and results of the tenfold cross-validation

	nRMSE	Mean nRMSE (CV)	SD nRMSE (CV)	nMAE	Mean nMAE (CV)	SD nMAE (CV)	95% theoretical LoA	95% empirical LoA
UK	0.076	0.077	0.0083	0.053	0.054	0.0046	± 0.25	− 0.20; 0.17
France	0.075	0.076	0.0062	0.052	0.053	0.0041	± 0.23	− 0.19; 0.17
Germany	0.079	0.080	0.0096	0.051	0.051	0.0037	± 0.19	− 0.16; 0.13

*nRMSE* normalized root mean square error, *nMAE* normalized mean absolute error, *LoA* levels of agreement, *CV* cross-validation, *SD* standard deviation, *UK* United Kingdom

**Fig. 2:**
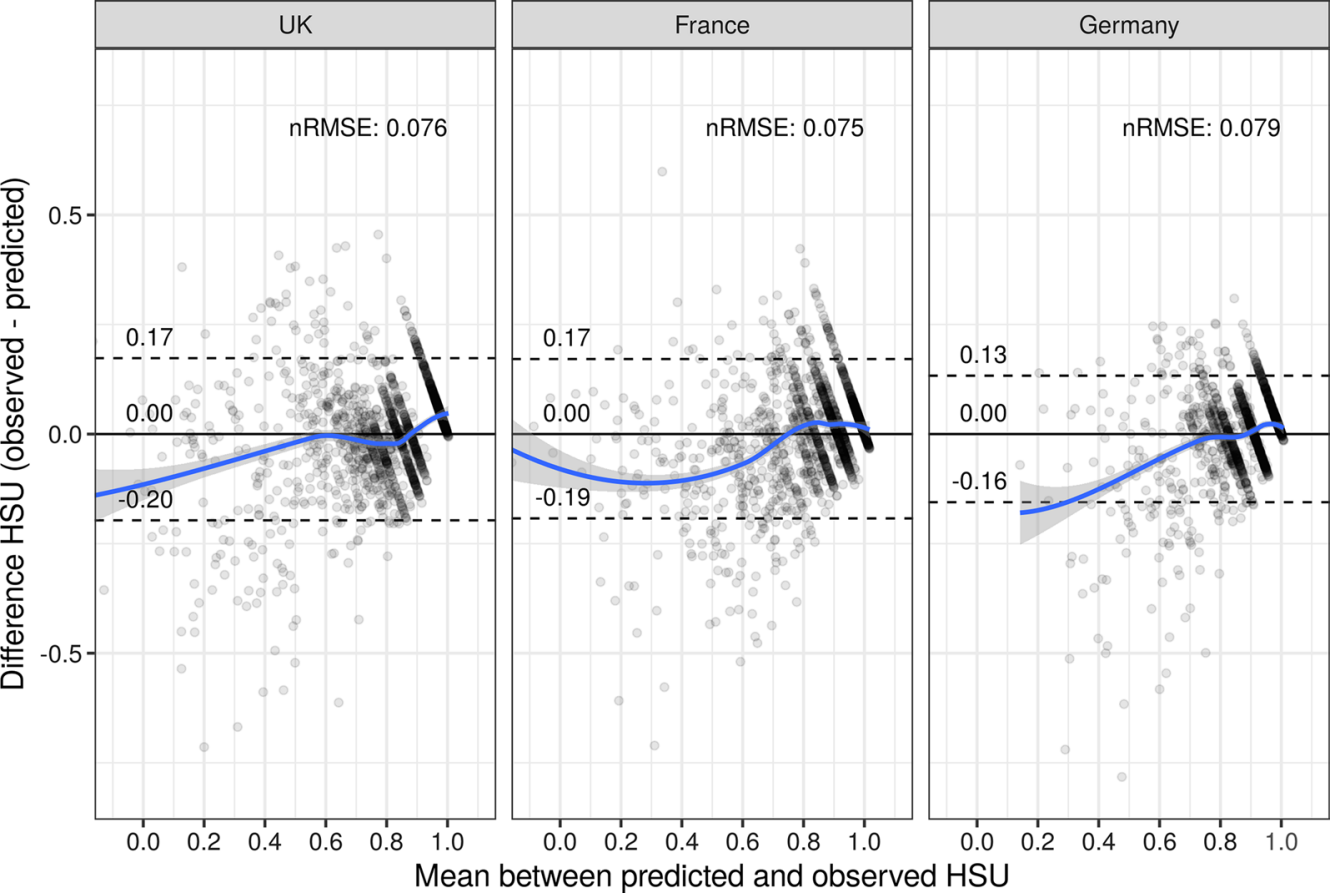
Bland–Altman plots of the predicted and observed health state utility scores for the UK, France, and Germany

The prediction performances of the final models along the HSU continuum are depicted in the Bland–Altman plots in Fig. 2. Note that especially in the German sample, there are not many respondents with low HSU (EQ-5D-5L crosswalk < 0.2). Furthermore, prediction performance appears to be slightly better for high levels of HSU (EQ-5D-5L crosswalk > 0.8) than for intermediate or low HSU.

### Impact of misspecified mapping functions on the prediction performance

The differences in the prediction performances between the applications of Revicki’s model versus our models are depicted in Table 3. The application of Revicki’s model to the European data would systematically underestimate the EQ-5D-5L crosswalk for the UK (− 0.10) and for France (− 0.09) but not for Germany. The prediction performance of Revicki’s model is the best in Germany, and the differences in the prediction performances between Revicki’s and our mapping functions are smaller in Germany than for the UK or for France, as indicated by the values of the nRMSE, nMAE, and empirical LoAs.

**Table 3:** The detrimental effect of using Revicki’s model to predict the EQ-5D-5L crosswalk from the PROMIS-29 for the United Kingdom, France, and Germany

	R^2^_adj_	ICC	Bias	nRMSE	nMAE	95% theoretical LoA	95% empirical LoA
*France*							
Revicki	0.61	0.78	− 0.09	0.112	0.072	− 0.38; 0.20	− 0.38; 0.08
Polynomial regression	0.72	0.85	0.00	0.075	0.052	± 0.23	− 0.19; 0.17
*Germany*							
Revicki	0.53	0.73	0.00	0.091	0.058	− 0.22; 0.22	− 0.18; 0.14
Polynomial regression	0.64	0.80	0.00	0.079	0.051	± 0.19	− 0.16; 0.13
*UK*							
Revicki	0.68	0.82	− 0.10	0.113	0.075	− 0.39; 0.19	− 0.39; 0.07
Polynomial regression	0.74	0.86	0.00	0.076	0.053	± 0.25	− 0.20; 0.17

*UK* United Kingdom, *adj* adjusted, *ICC* intraclass correlation coefficient, *nRMSE* normalized root mean squared error, *nMAE* normalized mean absolute error, *LoA* levels of agreement

**Fig. 3:**
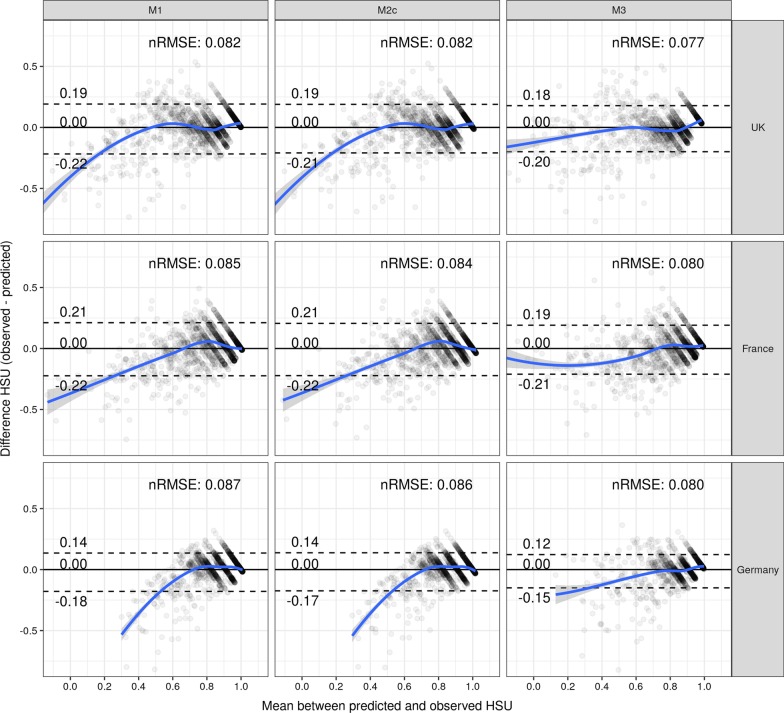
Incremental value of the country-specific regression coefficients, additional health domains, and higher-order effects for predicting the EQ-5D-5L crosswalk for the United Kingdom, France, and Germany

The last step was to investigate which factor was mainly responsible for the observed differences in the prediction performances between Revicki’s and our models. The results of the application of country-specific regression coefficients for the five health domains specified by Revicki (first alternative model; M1), the incorporation of sleep disturbance and/or participation (M2c), or the incorporation of quadratic and cubic trends into the five-domain model specified by Revicki (M3) are shown in Fig. 3. The average prediction performance (nRMSE_UK_ = 0.082, nRMSE_FRA_ = 0.085, and nRMSE_GER_ = 0.087) mainly improves by incorporating country-specific regression coefficients into the five health domain models specified by Revicki. However, neither this model (M1) nor the incorporation of sleep disturbance and/or participation (M2c) improves the prediction performance for low levels of HSU, but the incorporation of quadratic and cubic effects (M3) does improve the prediction performance for low levels of HSU. That is, overprediction of HSU is clearly reduced by adding these higher-order effects to the three regression equations.

## Discussion

### Summary of main findings

We developed optimal models for mapping the EQ-5D-5L crosswalk from the PROMIS-29 in the UK, France, and Germany. Furthermore, we showed that the incorporation of higher-order effects into the regression equations substantially reduced overestimation of low HSU. The EQ-5D-5L crosswalk can therefore now be predicted from PROMIS-29 in three major European countries for QALY in CUA for HTA assessments, enabling the use of PROMIS for economic evaluations in Europe. This is of practical importance since HTA agencies demand the EQ-5D-5L crosswalk as HSU for QALY and PROMIS is more frequently used in clinical, non-preference HRQoL. We believe our models are highly applicable achieving a good degree of precision, also in lower spectrums of health, while at the same time avoiding high complexity with a manageable number of predictors. Our results in terms of the nRMSE and nMAE perform very well compared to what is usually reported for mapping algorithms [17, 43–47].

The major comparator to our models is Revicki’s OLS linear US model, the only one predicting the EQ-5D-3L index value from PROMIS-29. All our models perform better in terms of R-squared and ICC while the LoA were comparable. Revicki did neither report MAE nor RMSE. Furthermore, Revicki’s uses the former version of the EQ-5D, the EQ-5D-3L with the US value set as target measure, while we use the EQ-5D-5L crosswalk value sets from the UK, France, and Germany, respectively. We demonstrated that the application of Revicki’s US model to European data will yield biased results, especially for poor health states. However, this model performs well in upper ranges of health. One might therefore consider using a foreign model with domestic data as a second-best option to predict the EQ-5D-5L crosswalk for QALY in CUA if a country-specific mapping algorithm is not available, especially in a group of healthier patients. This decision might make sense, for example, when using our German model for Austrian data in or using Revicki’s US model for Canadian data, since in both cases, cultural proximity can reasonably be assumed.

Apart from Revicki’s model predicting the EQ-5D-3L index value from PROMIS-29, there is also another model of his, predicting the EQ-5D-3L index value from PROMIS Global Health (GH) items, using linear regression in a US sample [19]. Thompson (2017) mapped PROMIS-GH to the EQ-5D-3L index value in a US sample applying linear and equipercentile equating, treating PROMIS-GH items as categorical variables [20]. So compared to our models, both models differ in respect of population, source measure, and target measure: They use the US value set for the EQ-5D-3L index value while we use the EQ-5D-5L crosswalk for the UK, France, and Germany, respectively. Thompson’s models additionally differs in the mapping method applied. In terms of R-squared, our model for Germany performs at least as good and our models for the UK and France perform better than both Revicki’s and Thompson’s PROMIS-GH models. In terms of MAE, all our models perform better. Despite Thompson’s the different method, low EQ-5D-3L index values where still overestimated [20]. Both studies did not report a RMSE.

Generally however, researchers should be aware that the consequences of working with a suboptimal mapping algorithm can be substantial: incremental cost-effectiveness ratio (ICER) of costs per QALY can differ between British pound sterling (GBP) 18,000 and GBP 32,000 depending on what mapping algorithm is used [48]. NICE has adopted a threshold of GBP 30,000 per QALY representing the public’s maximum additional willingness to pay for a new treatment or a new drug compared to the existing standard of care [49]. Consequently, imprecise mapping methods have a great impact on CUA in HTA assessments and consequently on what innovations are made available to patients.

### Strengths and limitations

This study was conducted using three large samples representative of the general population in three European countries. To ensure comparability, the sampling strategies were the same across countries. This strength of our study is directly related to its foremost weakness: Severe health states are not frequently observed in the general population, and the proposed models therefore rely on few observations for low HSU. Furthermore, our models allowed judgement of the incremental value of incorporating two additional health domains and higher-order effects for HSU prediction.

Finally, some authors have argued against OLS regression as a type of mapping method even though, as outlined above, it is the most widely used method. First, arguments against that method are due to the phenomenon of regression to the mean. Second, linear regression models tend to predict HSU score greater than one, which is a value that is impossible by definition of HSU [22]. In our study, the risk of predicting HSU values greater than one is circumvented by incorporation of non-linear trends.

### Directions for future research and the PROMIS preference score (PROPr) for QALYs

Our mapping functions should be confirmed to samples with a greater frequency of low HSU. Therefore, we are planning to replicate our findings with data collected from spine patients who were assessed before surgery. It would also be interesting whether regressing the EQ-5D dimensions on the PROMIS domain scores first and then calculating the EQ-5D-5L crosswalk from the regressed EQ-5D dimensions has incremental value [50].

PROMIS data can also be used to estimate a new preference-based HSU score: Hanmer developed the PROMIS Preference Score (PROPr) to compute HSU for QALYs directly from 7 PROMIS health domains: cognition, depression, fatigue, pain, physical function, sleep disturbance, and participation [51–55]. Note that these 7 PROMIS domains are not equivalent with those 7 domains from the PROMIS-29 profile (anxiety is missing in the PROPr, while cognition is missing in the PROMIS-29) [25, 53, 56, 57].

The PROPr could potentially be used instead of the EQ-5D-5L crosswalk in CUA. Since many European HTA authorities such as NICE specifically demand the use of the EQ-5D-5L crosswalk to measure HSU in CUA, mapping the PROMIS-29 to the EQ-5D-5L crosswalk will still be needed [49]. Also, as of December 2020, there is no PROPr value set for European preferences [53, 54].

## Conclusion

Our mapping functions can be used to predict the EQ-5D-5L crosswalk from the PROMIS-29 for CUA in HTA for the UK, France and Germany. The inclusion of polynomial regression terms decreases the prediction bias for lower HSU.

Our results support the assertion that mapping functions are country-specific. The application of Revicki’s model to the data collected in the three European countries leads to biased HSU estimates for the UK and France and to less precise estimates in all three countries. Estimation of country-specific regression coefficients for the five health domains identified by Revicki strongly improves the average prediction performance but does not remedy the overestimation of low HSU.

## Data Availability

Data is available on reasonable request.

## Appendix

See Table 4.

**Table 4:** Sociodemographic characteristics of the samples

Variable	Value	UK (%)	France (%)	Germany (%)	*p* value
Sex	Male	49.0	48.3	48.5	
	Female	51.0	51.7	51.5	0.918
Age	18–29	19.8	18.6	16.4	
	30–39	16.8	16.3	14.4	
	40–49	18.2	18.5	18.3	
	50–59	16.1	17.4	18.5	
	60–69	13.9	14.3	13.4	
	70 +	15.2	15.1	19.0	0.027
Education	Low	8.1	7.6	23.4	
	Medium	48.6	43.2	48.2	
	High	43.3	49.2	28.4	< 0.001
Occupation	Managers and professionals	21.2	10.8	12.5	
	Technicians, clerks, service workers	25.1	22.9	28.9	
	Workers, elementary occupations, armed forces	14.3	17.8	17.1	
	Inactive or unemployed	39.4	48.4	41.5	< 0.001
Income	Lower income	16.8	12.8	19.2	
	Lower-middle income	20.2	19.5	18.0	
	Higher-middle income	30.7	27.2	21.8	
	Higher income	21.0	24.4	24.2	
	Prefer not to answer	11.3	16.2	16.8	< 0.001

*UK* United Kingdom

## Abbreviations

3L: 3 levels
5L: 5 levels
BDI-2: Beck depression inventory version 2
BIC: Bayesian information criterion
CAT: Computerized adaptive testing
CES-D: Center for Epidemiologic Studies Depression Scale
CUA: Cost-utility analysis
CV: Cross validation
EQ-5D: EuroQoL 5 dimensions
FRA: France
GAD-7: Generalized anxiety disorder 7
GBP: British pound sterling
GER: Germany
GH: Global Health
HRQoL: Health-related quality of life
HSU: Health state utility
HTA: Health technology assessments
ICC: Intraclass correlation coefficient
ICER: Incremental cost-effectiveness ratio
LoA: Limits of agreement
MASQ: Mood and anxiety symptom questionnaire
NICE: National Institute of Health and Clinical Excellence
(n)MAE: (Normalized) mean absolute error
(n)RMSE: (Normalized) root mean square error
M: Mean
OLS: Ordinary least squares
PANAS: Positive and negative affect schedule
PHQ-9: Patient-health questionnaire-9
PROM: Patient reported outcome measure
PROMIS: Patient reported outcome measurement information system
PROMIS-29 v2.0: PROMIS profile 29 version 2.0
PROPr: PROMIS preference score
QALY: Quality-adjusted life years
RIM: Random iterative method
SD: Standard deviation
SG: Standard gamble
TTO: Time trade-off
UK: United Kingdom of Great Britain and Northern Ireland
US: United States of America
VAS: Visual Analogue Scale
